# Three hematologic/immune system-specific expressed genes are considered as the potential biomarkers for the diagnosis of early rheumatoid arthritis through bioinformatics analysis

**DOI:** 10.1186/s12967-020-02689-y

**Published:** 2021-01-06

**Authors:** Qi Cheng, Xin Chen, Huaxiang Wu, Yan Du

**Affiliations:** 1 Department of Rheumatology, the Second Affiliated Hospital of Zhejiang University School of Medicine, 88 Jiefang Road, Hangzhou, 310009 China; 2 Department of Clinic Medicine, The Second Affiliated Hospital of Zhejiang University School of Medicine, 88 Jiefang Road, Hangzhou, 310009 China

**Keywords:** Early rheumatoid arthritis, Tissue-specific expressed genes, Biomarker, Microarray, Bioinformatics analysis, RNA regulatory pathways

## Abstract

**Background:**

Rheumatoid arthritis (RA) is the most common chronic autoimmune connective tissue disease. However, early RA is difficult to diagnose due to the lack of effective biomarkers. This study aimed to identify new biomarkers and mechanisms for RA disease progression at the transcriptome level through a combination of microarray and bioinformatics analyses.

**Methods:**

Microarray datasets for synovial tissue in RA or osteoarthritis (OA) were downloaded from the Gene Expression Omnibus (GEO) database, and differentially expressed genes (DEGs) were identified by R software. Tissue/organ-specific genes were recognized by BioGPS. Enrichment analyses were performed and protein–protein interaction (PPI) networks were constructed to understand the functions and enriched pathways of DEGs and to identify hub genes. Cytoscape was used to construct the co-expressed network and competitive endogenous RNA (ceRNA) networks. Biomarkers with high diagnostic value for the early diagnosis of RA were validated by GEO datasets. The ggpubr package was used to perform statistical analyses with Student’s t-test.

**Results:**

A total of 275 DEGs were identified between 16 RA samples and 10 OA samples from the datasets GSE77298 and GSE82107. Among these DEGs, 71 tissue/organ-specific expressed genes were recognized. Gene Ontology (GO) and Kyoto Encyclopedia of Genes and Genomes (KEGG) enrichment analysis indicated that DEGs are mostly enriched in immune response, immune-related biological process, immune system, and cytokine signal pathways. Fifteen hub genes and gene cluster modules were identified by Cytoscape. Eight haematologic/immune system-specific expressed hub genes were verified by GEO datasets. GZMA, PRC1, and TTK may be potential biomarkers for diagnosis of early RA. NEAT1-miR-212-3p/miR-132-3p/miR-129-5p-TTK, XIST-miR-25-3p/miR-129-5p-GZMA, and TTK_hsa_circ_0077158- miR-212-3p/miR-132-3p/miR-129-5p-TTK might be potential RNA regulatory pathways to regulate the disease progression of early RA.

**Conclusions:**

This work identified three haematologic/immune system-specific expressed genes, namely, GZMA, PRC1, and TTK, as potential biomarkers for the early diagnosis and treatment of RA and provided insight into the mechanisms of disease development in RA at the transcriptome level. In addition, we proposed that NEAT1-miR-212-3p/miR-132-3p/miR-129-5p-TTK, XIST-miR-25-3p/miR-129-5p-GZMA, and TTK_hsa_circ_0077158-miR-212-3p/miR-132-3p/miR-129-5p-TTK are potential RNA regulatory pathways that control disease progression in early RA.

## Background

Rheumatoid arthritis (RA) is a common chronic autoimmune connective tissue disease that mainly involves the joints. The incidence of RA is 5 to 10 per 1000 people [1]. With the progression of the disease and the continuation of synovial inflammation, the involved joint tissue is gradually eroded. Eventually, RA leads to irreversible damage to the joint, which is a very large burden on individuals and society. However, early diagnosis and treatment of RA can effectively prevent disease progression, joint damage, and other complications in 90% of patients [2]. Therefore, the earlier a patient with RA is diagnosed, the less burden will be placed on the patient and society. At present, serum biomarkers used in the diagnosis of established RA are rheumatoid factor and anti-cyclic citrullinated peptide antibody [3]. However, early RA especially serum RF and anti-CCP antibody-negative is difficult to diagnose due to the lack of effective biomarkers. Studies have reported that some biomarkers, such as 14–3-3η autoantibodies and calprotectin, may be effective in the diagnosis of early RA [4–7]. However, because these biomarkers are not validated in prospective cohorts or the clinical relevance of them are unclear, they have not been used in clinical diagnosis. Therefore, it is vital to identify new and effective biomarkers for the early diagnosis and treatment of RA.

Currently, transcriptomic and microarray analyses have been widely used in a variety of diseases, including a variety of tumours and RA, to identify new biomarkers to improve diagnosis and treatment [8–12]. In addition, competitive endogenous RNA (ceRNA) networks can elucidate a new mechanism for promoting the development of the disease in a transcriptional regulatory network [13]. Through the combination of microarray and bioinformatics analyses, it is possible to explore potential key genes and pathway networks that are closely related to the development of diseases.

In the present study, we first downloaded microarray datasets for synovial tissue in RA or OA from the GEO database. After pre-processing and normalizing the data by the Robust Multiarray Average (RMA) method in R language, we identified DEGs based on the screening criteria and obtained the tissue/organ-specific expressed genes by the online tool BioGPS. Next, GO and KEGG enrichment analyses were performed by the Gene Set Enrichment Analysis (GSEA) software, R software clusterProfiler package, and online tool KEGG Orthology-Based Annotation System (KOBAS) 3.0. PPI network was constructed using the online tool STRING, and Cytoscape was used to identify cluster modules and hub genes related to RA. Then, target microRNAs (miRNAs) of selected hub genes were predicted by five online miRNA databases, and a co-expressed network was constructed with Cytoscape. Subsequently, we validated the selected hub genes using GEO datasets, and ceRNA networks were constructed based on prediction results of long noncoding RNAs (lncRNAs) and circular RNAs (circRNAs). This work provides insight into the mechanisms of disease development in RA at the transcriptome level and explores potential biomarkers for the early diagnosis and treatment of RA.

## Methods

### Microarray data acquisition

The GEO database was used to obtain microarray data for synovial tissue in RA or OA. Screening criteria included the following: (1) Homo sapiens Expression Profiling by array; (2) synovial tissue of RA or OA from joint synovial biopsies; (3) datasets contain more than five samples, (4) datasets contain complete information about the samples, (5) one biopsy sample per subject was analysed without replicates. Finally, two GPL570 datasets GSE77298 and GSE82107, which included 16 RA samples and 10 OA samples, were selected as test sets; three GPL96 datasets GSE55584, GSE55457, and GSE55235, which included 33 RA samples and 26 OA samples, and the GPL11154 GSE89408 dataset, which included 57 early RA samples, 95 established RA samples and 22 OA samples, were selected as the validation sets (Table 1).

**Table 1:** Information for selected microarray datasets

GEO accession	Platform	Samples		Source tissue	Age		Sex (male/female)		Attribute
		OA	RA		OA	RA	OA	RA	
GSE77298	GPL570	0	16	Synovium	–	–	–	–	Test set
GSE82107	GPL570	10	0	Synovium	–	–	–	–	Test set
GSE55584	GPL96	6	10	Synovium	73.7 ± 7.1	54.9 ± 12.9	0/6	3/7	Validation set
GSE55457	GPL96	10	13	Synovium	72.4 ± 5.9	60 ± 20	2/8	3/10	Validation set
GSE55235	GPL96	10	10	Synovium	–	–	–	–	Validation set
GSE89408	GPL11154	22	152 (57 early and 95 established)	Synovium	53.3 ± 19.8	55.1 ± 15	9/13	46/106	Validation set

Annotation: GPL570: [HG-U133_Plus_2] Affymetrix Human Genome U133 Plus 2.0 Array; GPL96: [HG-U133A] Affymetrix Human Genome U133A Array; GPL11154: Illumina HiSeq 2000 (Homo sapiens); *DEGs* differentially expressed genes

### Data normalization and identification of DEGs

The original files that were downloaded from the GEO database were pre-processed and normalized by the Robust Multiarray Average (RMA) method based on the R software (version 4.0.1) affy package. The limma package was used to conduct gene analysis of inter-sample differences, and multiple hypothesis testing and correction were conducted after p-value was obtained. The threshold value of p-value was determined by controlling False Discovery Rate (FDR), and the corrected p-value was adjusted p value (Q value)[14,15]. The screening criteria were log2 (fold change) > 1 or < -1 and adjusted p value (Q value) < 0.05.

### Heatmap and volcano plot analyses

To better visualize these DEGs, R software was used to make heatmaps and volcano plots. Heatmaps were made with the pheatmap package.

### Identification of tissue/organ-specific expressed genes

To understand the tissue/organ-specific expression of these DEGs, the online tool BioGPS (http://biogps.org/) was used to analyse the tissue distribution [16]. The screening criteria were as follows: (1) transcripts that mapped to a single organ system with an expression value of > 10 multiples of the median, and 2() second-most-abundant tissue’s expression was no more than a third as high [17]. The genes obtained by these criteria were considered to be tissue-specific genes.

### Enrichment analysis

GSEA was used to assess the distribution trend of the genes of a predefined set in the gene table to determine their contribution to the phenotype [18]. We downloaded GSEA_4.1.0 and c5: GO gene sets (c5.all.v7.1.symbols.gmt) for functional enrichment analyses. The R software clusterProfiler package was used to analyse the GO enrichment of DEGs, and a chord plot was created for the visualization of these enrichment results. KOBAS 3.0 is an online database widely used for gene/protein function annotation and pathway enrichment (http://kobas.cbi.pku.edu.cn/kobas3) [19]. KOBAS 3.0 was used for the KEGG pathway and Reactome enrichment analyses of DEGs. The significant enriched functions and pathways was selected with Q < 0.05. The Q value is the adjusted p value.

### Construction of the PPI network

The PPI network was constructed based on all DEGs by the online tool STRING (https://string-db.org/) with a filter condition (combined score > 0.4). Next, we downloaded the interaction information and optimized the PPI network with Cytoscape software (v3.8.0) for better visualization. Minimal Common Oncology Data Elements (MCODE) was used to identify significant gene clusters and obtain cluster scores (filter criteria: degree cut-off = 2; node score cut-off = 0.2; k-core = 2; max depth = 100). CytoHubba was used to identify significant genes in this network as hub genes [20]. We used five algorithms, namely Degree, Maximal Clique Centrality (MCC), Maximum Neighborhood Component (MNC), Density of Maximum Neighborhood Component (DMNC), and Clustering Coefficient, to calculate the top 30 hub genes [21,22]. Finally, all the results were intersected to obtain the final hub genes.

### Prediction of target miRNAs

We used five online miRNA databases, namely, RNA22, DIANA-micro T, miRWalk, miRDB, and miRcode, to predict target miRNAs of hub genes and selected miRNAs that were found in at least four databases as the target miRNAs. The messenger RNA (mRNA)-miRNA co-expressed network based on the relationship between mRNAs and miRNAs was constructed by using Cytoscape.

### Construction of ceRNA networks

StarBase (version 3.0) (http://starbase.sysu.edu.cn/index.php) was used to predict lncRNAs and circRNAs that interacted with the selected miRNAs [23]. The intersections of the predicted results were used as the target lncRNAs and circRNAs. CeRNA networks based on the interactions among mRNAs, miRNAs, and noncoding RNAs (ncRNAs) were constructed by using Cytoscape.

### Statistics analysis

The R software ggpubr package was used to perform statistical analyses, and the ggplot2 package was used to draw boxplots. Student’s t-test was used to compare the differences between the two groups. IBM SPSS Statistics 25 (SPSS, Inc., Chicago, IL, USA) was used to analyse the data and draw the ROC curve.

## Results

### Identification of DEGs

**Fig. 1:**
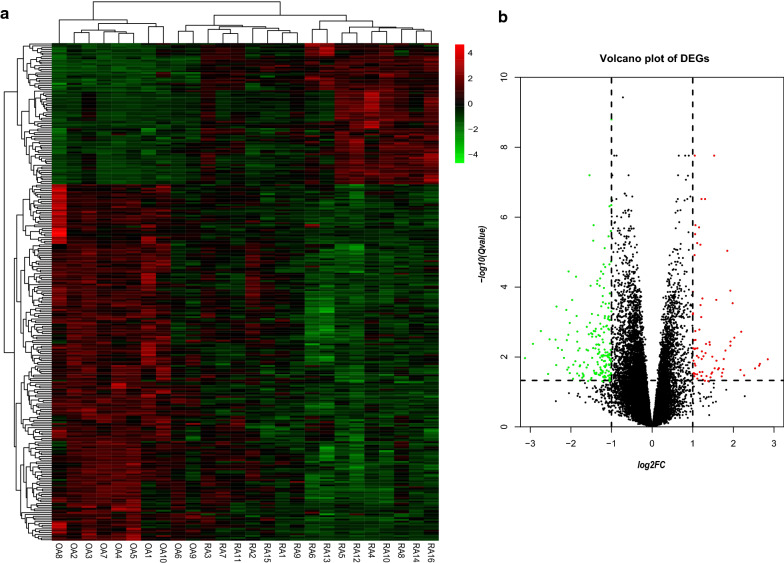
Identification of DEGs. **a** Heatmap of DEGs between the RA samples and the OA samples. Red rectangles represent high expression, and green rectangles represent low expression. **b** Volcano plot of DEGs between the RA samples and the OA samples. The red plots represent upregulated genes, the black plots represent nonsignificant genes, and the green plots represent downregulated genes.

The datasets GSE77298 and GSE82107, which included 16 RA samples and 10 OA samples, were selected to analyse and identify the DEGs. Compared with genes in the OA samples, we identified a total of 275 DEGs in the RA samples, which comprised 197 downregulated genes and 78 upregulated genes. Next, heatmap and volcano plot analyses were used to visualize these DEGs, which are shown in Fig. 1a, b.

### Identification of the tissue/organ-specific expressed genes

A total of 71 tissue/organ-specific expressed genes were identified by BioGPS (Table 2). We observed that most of these genes were specifically expressed in the haematologic/immune system (35/71, 49.29%). The second organ-specific expressed system was the nervous system, which included 13 genes (13/71, 18.31%). This was followed by the digestive system (7/71, 9.86%), respiratory system (4/71, 5.63%), circulatory system (4/71, 5.63%), and placenta (3/71, 4.22%). Finally, the endocrine system, genital system, and tongue, prostate, and adipose tissues had the lowest specific expressed genes (1/71, 1.41%).

**Table 2:** Distribution of tissue/organ-specific expressed genes identified by BioGPS

System/Organ		Genes	Counts
Haematologic/Immune			
Haematologic/Immune cells		PLA2G7, SLC50A1, T, MSC, MATK, PRKCD, CCR7, CYB561A3, P2RY8, CD3G, EMR2, NOV, BCL2A1, CD52, CD27, IL7R, TTK, MAP3K7CL, PNOC, FCGR1B, GZMB, GZMA, DLGAP5, TRBC1, MYOM2, CORO1A, PRC1, CEP55, CD3D, IER2, ITK, TNFRSF17	32
Immune organs		CXCL13, LCK, CD163	3
Nervous		PALM, KCND2, RASL10A, DACH1, STXBP1, DNM1, IL17D, PLP1, WRB, RCAN2, ZNF423, LRRN4CL, LPHN3	13
Digestive		GIPC2, AKR1B10, IGJ, ADAMDEC1, C6, TOX3, C15orf48	7
Respiratory		CHAD, MFAP4, CLDN5, LAMP3	4
Circulatory		ACTC1, CASQ2, LRRC2, CKMT2	4
Placenta		PVRL3, RHOBTB1, AGTR1	3
Endocrine		DUOX2	1
Genital		MLF1	1
Others			
Tongue		MAL	1
Prostate		PPAP2A	1
Adipose		HOXC10	1

**Table 3:** 15 hub genes identified by five algorithms of cytoHubba

Gene symbol	Description	log2FC	Q value	Regulation
*CXCL13*	C–X–C motif chemokine ligand 13	2.846	0.012	Up
*CD52*	CD52 molecule	1.928	0.004	Up
*GZMA*	Qranzyme A	1.753	0.022	Up
*CD27*	CD27 molecule	1.419	0.004	Up
*CEP55*	Centrosomal protein 55	1.264	0.01	Up
SKA3	Spindle and kinetochore associated complex subunit 3	1.215	3.03E−07	Up
IL21R	Interleukin 21 receptor	1.208	7E−04	Up
*DLGAP5*	DLG associated protein 5	1.172	0.027	Up
*PRC1*	Protein regulator of cytokinesis 1	1.112	0.042	Up
EOMES	eomesodermi	1.108	0.031	Up
*TTK*	TTK protein kinas	1.065	0.006	Up
CDCA8	Cell division cycle associated 8	1.049	1.74E−08	Up
UHRF1	Ubiquitin-like with PHD and ring finger domains 1	1.048	0.016	Up
KIAA0101	PCNA clamp associated factor	1.017	0.013	Up
FNBP1L	Formin binding protein 1 like	−1.325	0.004	Down

Annotation: FC: fold change, Q value: adjust P-value. The gene symbol in bold indicates eight hematologic/immune system-specific expressed hub genes

### Enrichment analysis

**Fig. 2:**
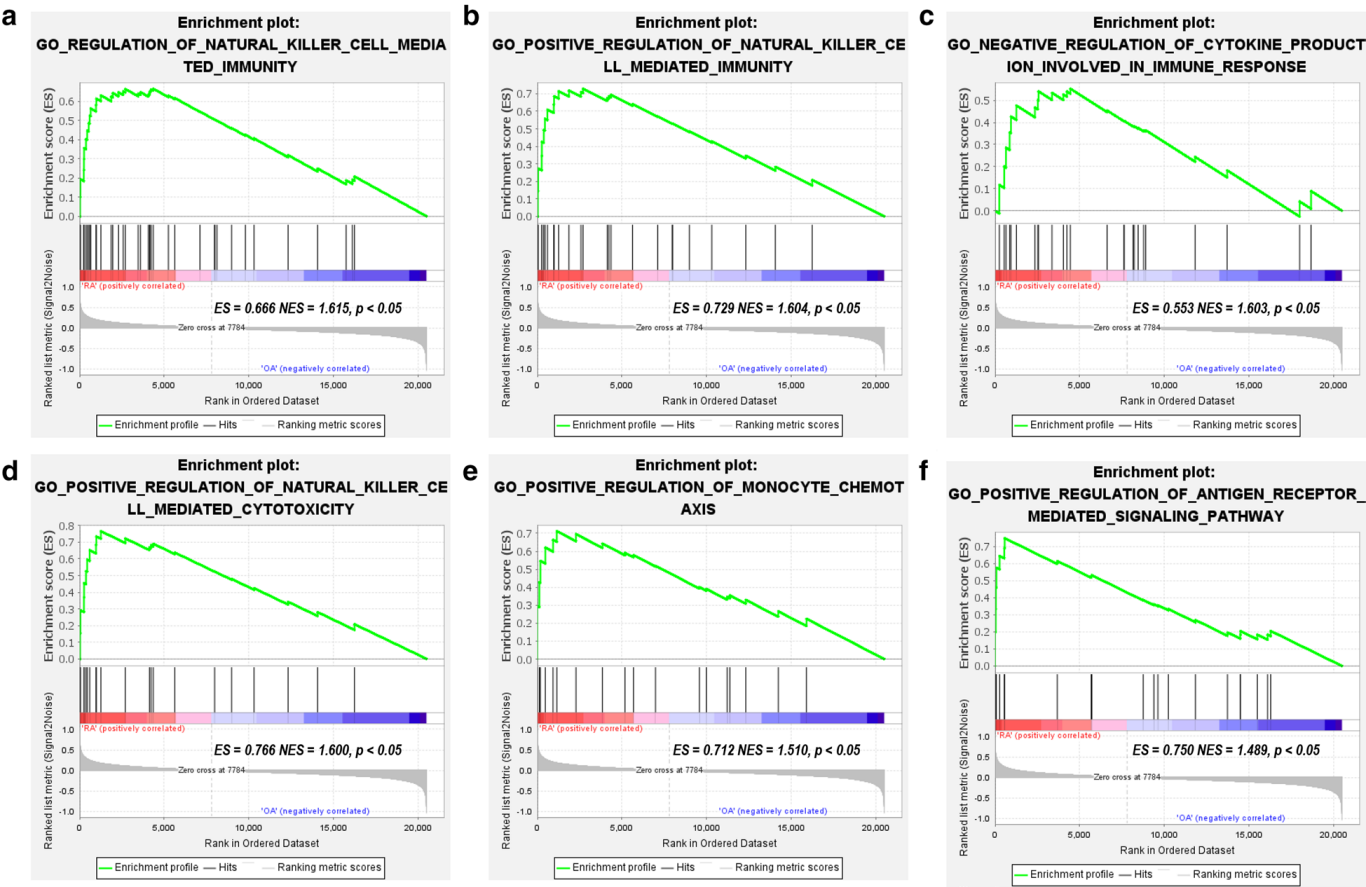
GSEA plot showing most enriched immune-related gene sets in the RA group and OA group. The c5: GO gene set was used to perform the GO enrichment analysis of the expression profile at the overall level. **a** The most significant enriched immune-related gene set was regulation of natural killer cell mediated immunity (ES = 0.666, NES = 1.615, p < 0.05). **b** The second significant enriched immune-related gene set was positive regulation of natural killer cell medicated immunity (ES = 0.729, NES = 1.604, p < 0.05). **c** The third significant enriched immune-related gene set was negative regulation of cytokine production involved in immune response (ES = 0.553, NES = 1.603, p < 0.05). **d** The fourth significant enriched immune-related gene set was positive regulation of natural killer cell mediated cytotoxicity (ES = 0.766, NES = 1.600, p < 0.05). **e** The fifth significant enriched immune-related gene set was positive regulation of monocyte chemotaxis (ES = 0.712, NES = 1.510, p < 0.05). F. The sixth significant enriched immune-related gene set was positive regulation of antigen receptor mediated signaling pathway (ES = 0.750, NES = 1.489, p < 0.05). The screening criteria for significant gene sets were p < 0.05 and Q < 0.25. ES: enrichment score; NES: normalized enrichment score; Q: also named False Discovery Rates (FDR)or adjust p value

The GSEA software, R software clusterProfiler package, and online tool KOBAS 3.0 were used for functional and pathway enrichment analyses. First, we uploaded the expression profile of all genes in the RA and OA samples to GSEA, and the c5: GO gene set was used to perform the GO enrichment analysis of the expression profile at an overall level. The screening criterion for significant gene sets was p < 0.05 and Q < 0.25. We observed that most of the enriched gene sets were related to the innate immune cell-mediated immune response, immune-related biological processes, and pathways (Fig. 2).

**Fig. 3:**
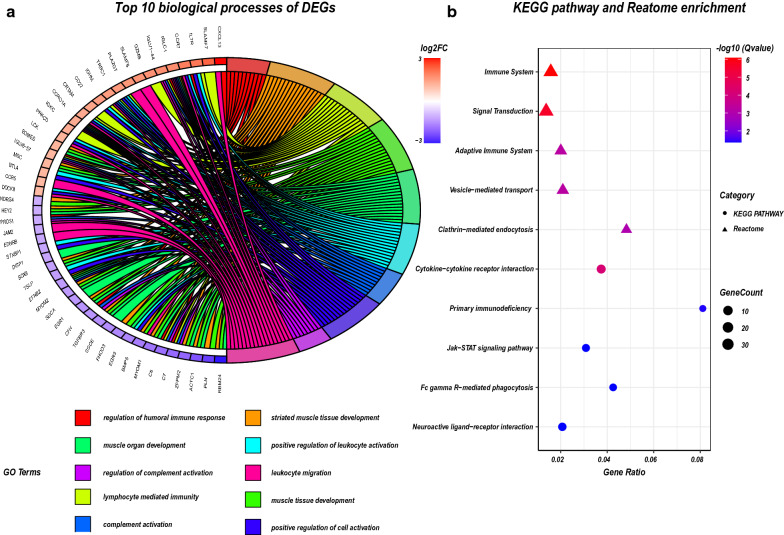
GO, KEGG pathway, and Reactome enrichment analyses of DEGs. **a** The chord plot showing the top 10 enriched biological processes of DEGs. Compared with OA, RA patients have stronger immune cells and complement system activation in the synovium, such as leukocyte and lymphocyte. **b** The bubble plot showing the most enriched KEGG and Reactome pathways of DEGs. The most significant KEGG pathways involved cytokines and their signaling pathways, while the most significant Reactome pathways were immune system and signal transduction. These indicate that the immune signal is more strongly activated in RA synovium, which is consistent with the clinical manifestations (such as arthritis). The screening criteria for significant enriched biological processes and pathways were Q < 0.05. The Q value is the adjusted p value

Next, the R software clusterProfiler package and KOBAS 3.0 were used to perform GO, KEGG pathway, and Reactome enrichment analyses of DEGs, respectively. The KEGG pathway is more comprehensive and contains more genes; while the Reactome pathway has more specific functions and focuses more on biochemical reactions[24]. We will observe the enrichment pathway of DEGs from multiple perspectives. GO enrichment analysis of DEGs also revealed that the immune response in RA samples was stronger than that in OA samples, and this included the regulation of humoral immune response, complement activation, leukocyte activation, and migration. The top 10 biological processes were selected based on a Q value < 0.05 and were drawn in a chord plot (Fig. 3a). KEGG pathway enrichment analysis showed that DEGs were enriched in cytokine-cytokine receptor interaction, primary immunodeficiency, JAK-STAT signalling pathway, Fc gamma R-mediated phagocytosis, and neuroactive ligand-receptor interaction. Reactome enrichment analysis showed that DEGs were mostly enriched in the immune system and signal transduction. According to Q value < 0.05, we selected the top five KEGG pathways and the top five Reactome terms and showed them in a bubble plot (Fig. 3b).

### PPI network analysis, MCODE cluster modules and hub gene identification

**Fig. 4:**
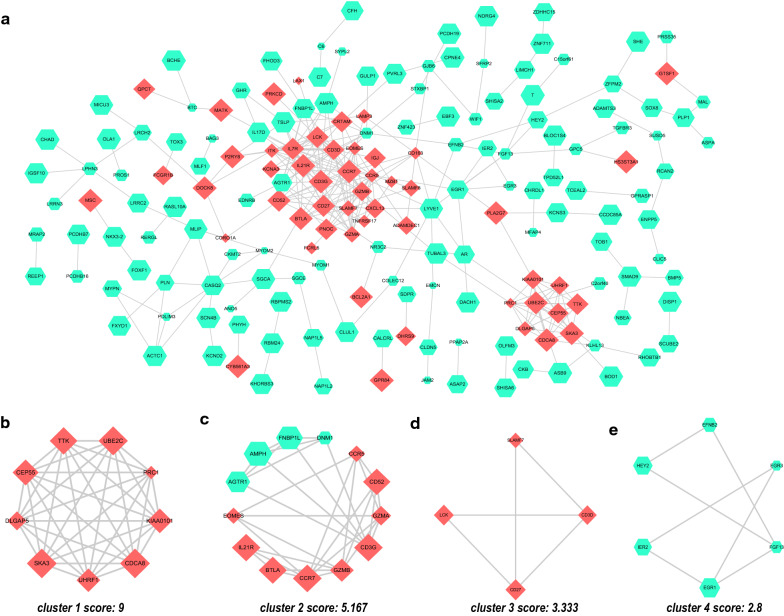
PPI network of DEGs and four cluster modules extracted by MCODE. **a** The interaction network between proteins coded by DEGs was comprised of 187 nodes and 307 edges. Each node represents a protein, while each edge represents one protein–protein association. Red diamonds represent the upregulated genes, and green hexagons represent the downregulated genes. The smaller the value of Q is, the larger the shape size. Four cluster modules extracted by MCODE. Cluster 1 (**b**) had the highest cluster score (score: 9, 9 nodes and 36 edges), followed by cluster 2 (**c**) (score: 5.167, 13 nodes and 31 edges), cluster 3 (**d**) (score: 3.333, 4 nodes and 5 edges), and cluster 4 (**e**) (score: 2.8, 6 nodes and 7 edges)

The interaction network between proteins coded by DEGs, which was comprised of 187 nodes and 307 edges, was constructed by STRING and visualized by Cytoscape (Fig. 4a). The MCODE plugin was used to identify gene cluster modules. In this network, we identified four modules, which are shown in Fig. 4b-e, according to the filter criteria. Cluster 1 had the highest cluster score (score: 9, 9 nodes and 36 edges), followed by cluster 2 (score: 5.167, 13 nodes and 31 edges), cluster 3 (score: 3.333, 4 nodes and 5 edges), and cluster 4 (score: 2.8, 6 nodes and 7 edges). Next, we used the cytoHubba plugin to identify hub genes. Fifteen hub genes were identified by intersecting the results from the five algorithms of cytohubba including Degree, MCC, MNC, DMNC, and Clustering Coefficient [20]. These hub genes with detailed information are shown in Table 3. These genes are the most important genes in PPI network and may play an important role in the pathogenesis of RA. Additionally, GO and KEGG enrichment analyses showed that DEGs were mainly enriched in immune-related biological processes and pathways. As the most common autoimmune disease, a better understanding of immune-related mechanisms in RA is an important part of current research. The discovery of genes specifically expressed by the immune system in RA synovium may contribute to the discovery of key targets in the pathogenesis of RA. Therefore, we intersected 15 hub genes and genes specifically expressed in the haematologic/immune system. Ultimately, we obtained eight haematologic/immune system-specific expressed hub genes, includin–g CD52, CD27, TTK, GZMA, DLGAP5, PRC1, CEP55, and CXCL13 (Table 3, in italics).

### Prediction of target miRNAs and construction of the co-expressed network

**Fig. 5:**
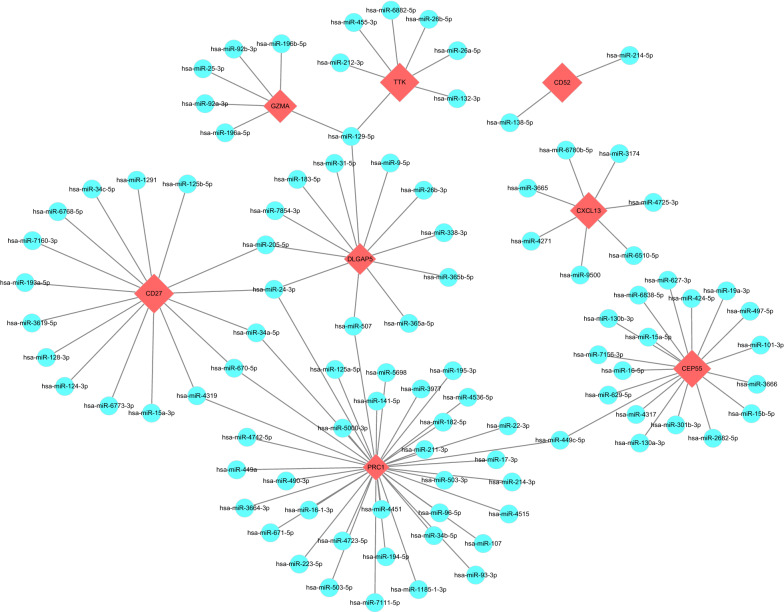
A co-expressed network of mRNAs and target miRNAs. The mRNA-miRNA co-expressed network was constructed by Cytoscape including 103 nodes and 105 edges**.** PRC1 has the most target miRNAs (37), while CD52 has only 2 target miRNAs. One node represents a mRNA or miRNA, while one edge represents one interaction of mRNA and miRNA. Red diamonds represent the hub genes, and blue circles represent miRNAs

We used five online miRNA databases to predict target miRNAs of hub genes. Finally, we obtained 95 target miRNAs of 8 specifically expressed hub genes and determined 105 mRNA-miRNA pairs. According to the prediction results, a co-expressed network of mRNAs and miRNAs, which comprised 103 nodes and 105 edges, was constructed by Cytoscape (Fig. 5).

### Verification of the 8 specifically expressed hub genes by 4 datasets from the GEO database

**Fig. 6:**
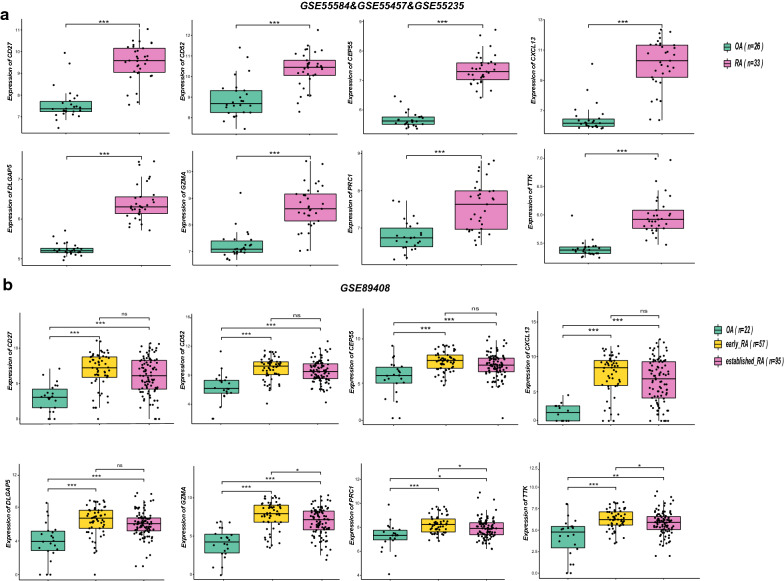
Verification of the 8 specifically expressed hub genes by 4 datasets of the GEO database. **a** Verification by three GPL96 datasets: GSE55584, GSE55457 and GSE55235. Compared with OA samples, all hub genes are upregulated in RA samples with significance. **b** Verification by the GPL11154 GSE89408 dataset. ***: p < 0.001, **: p < 0.01, *: p < 0.05, ns: no significant difference. Compared with OA samples, all hub genes are upregulated in RA samples with significance. Compared with established RA samples, GZMA, PRC1, and TTK were upregulated in early RA samples, while others have no significance

Three GPL96 datasets, namely, GSE55584, GSE55457 and GSE55235, which included 33 RA samples and 26 OA samples, and the GPL11154 GSE89408 dataset, which included 57 early RA samples, 95 established RA samples and 22 OA samples were selected to verify the 8 specifically expressed hub genes. The R software ggplot2 package and ggpubr package were used to draw boxplots and perform Student’s t-test statistical analyses. Consistent with our predictions, the mRNA expression levels of the 8 specifically expressed hub genes in the RA samples were significantly increased compared with those in the OA samples (p < 0.01) (Fig. 6a, b). In addition, we observed that the mRNA expression levels of GZMA, PRC1, and TTK in the 57 early RA samples were significantly increased compared with those in the 95 established RA samples (p < 0.05) (Fig. 6b).

### ROC curve of the 8 specifically expressed hub genes in early RA samples and established RA samples

**Fig. 7:**
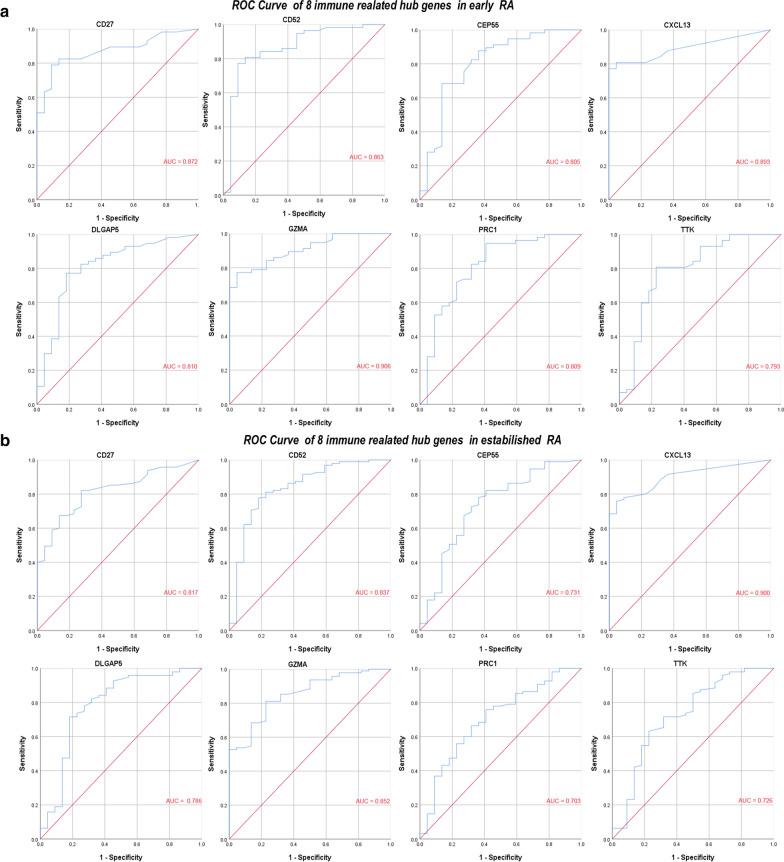
ROC curve of the 8 specifically expressed hub genes. **a** ROC curve of the 8 specifically expressed hub genes in early RA samples. **b** ROC curve of the 8 specifically expressed hub genes in established RA samples. *AUC* area under the ROC curve

We used IBM SPSS Statistics 25 to analyse the 8 specifically expressed hub genes expression profiles of OA samples, early RA samples, and established RA samples and draw the ROC curves. Area under the curve (AUC) is an indicator combining sensitivity and specificity, which can describe the intrinsic effectiveness of diagnostic tests [25]. Compared to OA samples, these 8 specifically expressed hub genes have higher diagnostic value both in the early RA samples and in the established RA samples. Among them, GZMA has the highest diagnostic value (AUC: 0.906) in the early RA samples, while CXCL13 has the highest diagnostic value (AUC: 0.900) in the established RA samples. The diagnostic value of other genes are follows: in early RA samples, CXCL13 (AUC: 0.893), CD27 (AUC: 0.872), CD52 (AUC: 0.863), DLGAP5 (AUC: 0.810), PRC1 (AUC: 0.809), CEP55 (AUC: 0.805), TTK (AUC: 0.793) (Fig. 7a), while in established RA samples, GZMA (AUC: 0.852), CD27 (AUC: 0.817), CD52 (AUC: 0.837), DLGAP5 (AUC: 0.786), PRC1 (AUC: 0.703), CEP55 (AUC: 0.731), TTK (AUC: 0.726) (Fig. 7b). Due to their good diagnostic performance in both early RA and established RA, we combined with their expression levels in early RA and established RA to identify better biomarkers. Compared with established RA, GZMA, PRC1 and TTK were up-regulated in early RA with statistical significance (Fig. 6b). Therefore, we hypothesize that GZMA, PRC1 and TTK may be biomarkers for early diagnosis of RA based on our present samples.

### Prediction of target ncRNAs and construction of ceRNA networks

**Fig. 8:**
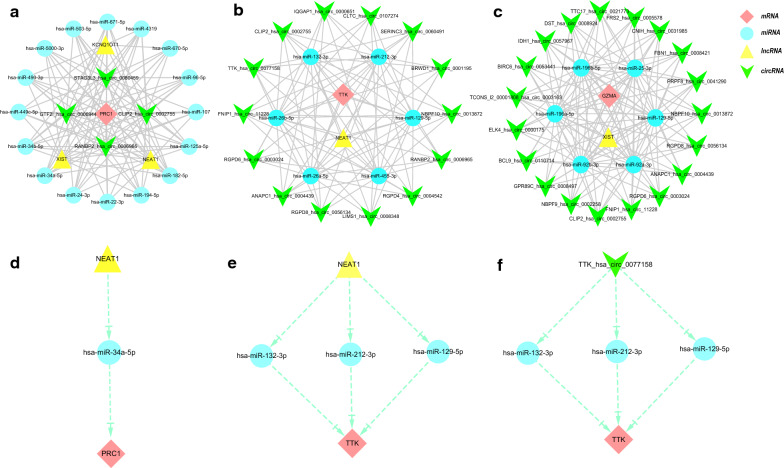
Three ceRNA networks of PRC1, TTK, and GZMA and the potential RNA regulatory pathways. **a** ceRNA network of PRC1. **b** ceRNA network of TTK. **c** ceRNA network of GZMA. **d** NEAT1-miR-212-3p/miR-132-3p/miR-129-5p-TTK. **e** XIST-miR-25-3p/miR-129-5p-GZMA. **f** TTK_hsa_circ_0077158-miR-212-3p/miR-132-3p/miR-129-5p-TTK. Red diamonds represent the hub genes, blue circles represent miRNAs, yellow triangle represents lncRNAs, and the V represents the circRNA

miRNAs are well known to induce gene silencing and downregulate gene expression by binding mRNAs. However, its upstream molecules, circRNAs, and lncRNAs, can affect the function of miRNA by combining miRNA response elements, thus upregulating gene expression. This interaction between RNAs is called a ceRNA network [13]. Next, we used the online database Starbase 3.0 to predict the lncRNAs and circRNAs that interact with the selected miRNAs. The screening criteria were: mammalian, human h19 genome, strict stringency (> = 5) of CLIP-Data, and with or without degradome data. We chose the ncRNAs that exist in most of the prediction results of miRNAs as our predicted lncRNAs and circRNAs. In addition, since a transcript has multiple circRNA shear sites in the prediction results of the Starbase database, we selected the circRNA with the most samples and highest score in the circBase database as the target circRNA. Finally, we obtained 3 target lncRNAs and 4 target circRNAs of the target miRNAs of PRC1; 1 target lncRNA and 19 target circRNAs of the target miRNAs of GZMA; and 1 target lncRNA and 14 target circRNAs of the target miRNAs of TTK. Three ceRNA networks based on the prediction results were constructed and illustrated by Cytoscape and are shown in Fig. 8a-c. Subsequently, according to the ceRNA hypothesis, we conducted a literature search and selected four reported downregulated miRNAs and an upregulated lncRNA in RA and upregulated lncRNA in another autoimmune disease, Sjogren's syndrome, for further analysis. We propose that NEAT1-miR-212-3p/miR-132-3p/miR-129-5p-TTK (Fig. 8d) and XIST-miR-25-3p/miR-129-5p-GZMA (Fig. 8e) might be potential RNA regulatory pathways to regulate the disease progression of early RA. Regarding the prediction results of circRNAs, we found a circRNA (TTK_hsa_circ_0077158) predicted by target miRNAs of TTK, and its target is TTK. Hence, we propose the following circRNA-miRNA-mRNA pathway: TTK_hsa_circ_0077158-miR-212-3p/miR-132-3p/miR-129-5p-TTK (Fig. 8f); it might be a key regulatory pathway in the pathogenesis of early RA.

## Discussion

The main characteristic of RA is chronic synovial inflammation, which leads to erosion and damage of joints. Early diagnosis and treatment of RA will effectively prevent joint damage and improve quality of life. However, early RA is difficult to diagnose due to the lack of effective biomarkers. It is crucial to identify new and effective biomarkers for the early diagnosis and treatment of RA.

In our study, we identified 275 DEGs, including 71 tissue/organ-specific expressed genes, by comparing genes expressed in RA and OA samples. GO enrichment analysis of all genes and DEGs indicated that the immune responses, such as the immune cell-mediated immune response and the regulation of humoral immune response, were stronger in RA samples than in OA samples. KEGG pathways that were enriched included cytokine-cytokine receptor interaction, primary immunodeficiency, JAK-STAT signalling pathway, Fc gamma R-mediated phagocytosis, and neuroactive ligand-receptor interaction. Reactome enrichment analysis also showed that DEGs were mostly enriched in the immune system and signal transduction. GO, KEGG and Reactome enrichment analysis all showed that RA synovial membrane had strong immune activation and signal transduction, which was the main cause of RA synovial inflammation, leading to arthritis and arthralgia. It is well known that arthritis and arthralgia are the main clinical manifestations of RA[1].

After the hub genes that were screened by the PPI network were validated using the GEO datasets, we identified eight haematologic/immune system-specific expressed genes. ROC curve analysis suggests that these genes have high diagnostic value for both early RA and established RA. Combined with their expression levels in early RA and established RA, GZMA, PRC1 and TTK were up-regulated in early RA with statistical significance (p < 0.05). Therefore, we hypothesize that GZMA, PRC1 and TTK may be biomarkers for early diagnosis of RA based on our present samples. In addition, we constructed an mRNA-miRNA co-expression network and ceRNA networks to elucidate the pathogenesis of RA at the transcriptome level.

GZMA, a member of the serine protease family, is secreted by cytotoxic cells such as cytotoxic T cells and natural killer (NK) cells and plays an important role in cell death, cytokine processing, and inflammation [26,27]. Several studies have reported that compared with the expression level of GZMA in OA patients, the expression level of GZMA increases in plasma, synovial tissues, and synovial membranes in patients with RA [28,29]. This indicates that GZMA plays a significant role in the pathogenesis of RA. Consistent with this research, our study found that GZMA was upregulated in the synovial membrane of RA, especially in early RA. In addition, the ROC curve of GZMA indicated that it has a very high diagnostic value in early RA (AUC = 0.906). We considered GZMA a very effective biomarker for the diagnosis of early RA.

PRC1 (also called ASE1), a human mitotic spindle-associated CDK substrate protein, is a key regulator of cell division [30]. According to BioGPS, PRC1 is specifically expressed in early erythrocytes, endotheliocytes, and B lymphocytes. At present, PRC1 has not been reported in RA-related studies. However, in our study, PRC1 was upregulated in the synovial membrane of RA, especially in early RA. Compared with OA, synovial inflammation and hyperplasia are marked in RA. In addition, it has been reported that the metabolic level of the synovial membrane is elevated, similar to that of tumour tissue [31]. These results all reflect the increased proliferation of cells like synovial fibroblasts and macrophages in the synovial membrane of RA to some extent [32,33]. Therefore, PRC1 may play an important role in the proliferation of synovial cells and the disease progression of RA.

TTK (also called MPS1 and CT96), which encodes a dual specificity protein kinase that phosphorylates a variety of amino acids such as tyrosine and serine, is related to cell proliferation [34]. Similar to PRC1, TTK is also highly specifically expressed in early red blood cells and endothelial cells. A study by H Ah-Kim et al. reported that tumour necrosis factor-alpha (TNF-α) can increase TTK expression in human articular chondrocytes [35], suggesting that TTK is regulated by TNF-α in some biological processes. We know that TNF-α plays a very significant role in the pathogenesis of RA [36]. Thus, we hypothesized that TTK plays an important role in synovial cell proliferation and TNF-α-mediated pathogenesis. In addition, we identified that TTK was highly expressed in the synovial membrane of RA and has a high diagnostic value in early RA (AUC = 0.793). We considered TTK as a novel and effective biomarker for the diagnosis of early RA.

Furthermore, target miRNAs and the target lncRNAs and circRNAs of these miRNAs were predicted for GZMA, PRC1, and TTK, and a ceRNA network was constructed with Cytoscape. This network reveals the mechanism by which selected genes are regulated at the transcriptome level. According to the ceRNA hypothesis, we performed a literature search to select downregulated miRNAs in RA for further analysis. Among the target miRNAs of GZMA, PRC1, and TTK, the expression of the following miRNAs was downregulated in RA: miR-129-5p (in RA synovial tissue and synovial fibroblasts), miR-132-3p (in RA synovial fibroblasts), miR-212-3p (in RA synovial tissue and synovial fibroblasts), and miR-25-3p (in peripheral blood mononuclear cells) [37–40]. In addition, it has been reported that the lncRNA NEAT1 is upregulated in peripheral blood mononuclear cells of patients with RA [41]. Therefore, we propose that NEAT1-miR-212-3p/miR-132-3p/miR-129-5p-TTK might be a potential RNA regulatory pathway to regulate the disease progression of early RA. Additionally, although lncRNA XIST has not been reported in RA, it has been reported to be upregulated in another autoimmune disease, Sjogren's syndrome [42]. We hypothesize that XIST-miR-25-3p/miR-129-5p-GZMA has an important regulatory role in RA. Regarding the prediction results of circRNAs, we found a circRNA (TTK_hsa_circ_0077158) predicted by target miRNAs of TTK, and its target was TTK. Hence, we proposed a circRNA-miRNA-mRNA pathway: TTK_hsa_circ_0077158-miR-212-3p/miR-132-3p/miR-129-5p-TTK; it might be a key regulatory pathway in the pathogenesis of early RA. Of course, in our study, the sample size for analysis and verification is relatively small. Therefore, future studies need to increase the sample size and conduct prospective cohort studies to further confirm our views.

## Conclusions

Our work identified three haematologic/immune system-specific expressed genes, GZMA, PRC1, and TTK, as potential biomarkers for the early diagnosis and treatment of RA and provided insight into the mechanisms of disease development in RA at the transcriptome level. In addition, we propose that NEAT1-miR-212-3p/miR-132-3p/miR-129-5p-TTK, XIST-miR-25-3p/miR-129-5p-GZMA, and TTK_hsa_circ_0077158- miR-212-3p/miR-132-3p/miR-129-5p-TTK are potential RNA regulatory pathways that control disease progression in early RA.

## Data Availability

The [GSE datasets] data that support the findings of this study are available in the GEO database (https://www.ncbi.nlm.nih.gov/geo/) with the following data accession identifier(s): GSE77298, GSE82107, GSE55584, GSE55457, GSE55235, and GSE89408.

## Abbreviations

RA: Rheumatoid arthritis
OA: Osteoarthritis
GEO: Gene Expression Omnibus
DEGs: Differentially expressed genes
FDR: False Discovery Rate
GO: Gene Ontology
KEGG: Kyoto Encyclopedia of Genes and Genomes
ceRNA: Competitive endogenous RNA
GSEA: Gene Set Enrichment Analysis
RMA: Robust Multiarray Average
KOBAS: KEGG Orthology-Based Annotation System
PPI: Protein–protein interaction
MCC: Maximal Clique Centrality
MNC: Maximum Neighborhood Component
DMNC: Density of Maximum Neighborhood Component
ROC: Receiver operating characteristic
AUC: Area under the ROC curve
ncRNAs: Noncoding RNAs
